# Beneath the Antarctic sea‐ice: Fine‐scale analysis of Weddell seal (*Leptonychotes weddellii*) behavior and predator–prey interactions, using micro‐sonar data in Terre Adélie

**DOI:** 10.1002/ece3.10796

**Published:** 2023-12-11

**Authors:** Adélie Antoine, Sara Labrousse, Pauline Goulet, Mathilde Chevallay, Joris Laborie, Baptiste Picard, Christophe Guinet, David Nerini, Jean‐Benoît Charrassin, Karine Heerah

**Affiliations:** ^1^ Laboratoire d'Océanographie et du Climat: Expérimentations et approches numériques (LOCEAN), UMR 7159 Sorbonne‐Université, CNRS, MNHN, IRD, IPSL Paris France; ^2^ Centre d'Etudes Biologiques de Chizé, CNRS‐La Rochelle Université, UMR 7372 Villiers‐en‐Bois France; ^3^ Department of Biology Aarhus University Aarhus Denmark; ^4^ Bretagne Vivante Brest France; ^5^ Mediterranean Institute of Oceanography, Pytheas Institute, UMR 7294 Marseille France; ^6^ France Energie Marine Plouzané France

**Keywords:** bio‐logging, diving, foraging behavior, predator–prey interaction, sonar tags, Weddell seal

## Abstract

Lactation is the most energy‐demanding event in mammals' reproduction. In pinnipeds, females are the only food providers to the young and have developed numerous behavioral and physiological lactation strategies, from capital‐breeding to income‐breeding. Lactating females' fine‐scale foraging strategy, and precise understanding of how females supplement their pup's needs as well as their own are important to understand the species' ecology and energetic balance. Polar pinnipeds, inhabiting extreme environments, are sensitive to climate change and variability, understanding their constraints and foraging strategy during lactation is therefore important. In 2019, three sonar tags were deployed on lactating Weddell seals in Terre Adélie (East Antarctica) for 7 days, to study fine‐scale predator–prey interactions. Feeding activity was mostly benthic, reduced, central‐placed, and spatially limited. Females spent most of their time hauled‐out. A total of 331 prey capture attempts (PrCAs) were recorded using triaxial acceleration data, with 125 prey identified on echograms (5 cm, acoustic size). All PrCAs occurred on the seafloor, shallower than usual records (mean depth of 88 m, vs 280 m after their molt). We also found that they only fed in three of the five identified dive shapes, during the ascent or throughout the dive. Half of the prey were reactive to the seal's approach, either leaving the seafloor, or escaping just above the seafloor, suggesting that the seals hunt by chasing them from the seabed. Seals continuously scanned the area during the approach phase, evoking opportunistic foraging. Our results provide additional evidence that Weddell seal forage during lactation, displaying a mix of capital‐breeding and income‐breeding strategies during this period of physiological stress. This work sheds light on previously unexplored aspects of their foraging behavior, such as shallow water environments, targeting benthic prey, generally focusing on single prey rather than schools, and evidence of visual scanning through observed head movements.

## Graphical abstract

We describe fine‐scale lactating Weddell seals' foraging ecology, providing high‐resolution diving behavior and associated prey encounters using new sonar tags. Females displayed a mix of capital and income‐breeding strategies. Feeding activity was mostly benthic (contrary to non‐lactation period), reduced, and spatially limited. Dives of specific shapes were dedicated to hunting, while they also performed numerous dives without encountering prey. This period of feeding pressure might put them at high risk regarding their response to environmental variability.

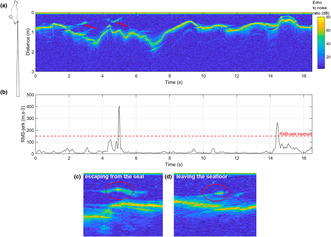

## 1 INTRODUCTION

Feeding strategies have evolved to deal with high energy requirements during lactation and brood care, both in terrestrial and marine mammals: Offspring are dependent on the mother during lactation, and females must maintain their own metabolism (Boness & Bowen, 1996; Borras‐Chavez et al., 2022; Clutton‐Brock et al., 1989; Costa et al., 1986; Gittleman & Thompson, 1988; Naya et al., 2008; Sapriza, 2019; Williams et al., 2007). Individuals' fitness is highly dependent on maternal allocation, that is, the energy invested by females in reproduction. One of the main aspects of females' reproductive success is their ability to store the energy needed for gestation and for provisioning their offspring. A distinction has been described by Drent and Daan (1980), between breeding females energy management strategies: “income” (the female feeds during lactation) and “capital” (the female relies on her body reserve and fasts during lactation) breeding. In pinnipeds, females are the only providers of food to the young, having to find the resources needed to produce milk (Boness & Bowen, 1996). Species of this taxa have evolved by developing numerous behavioral and physiological lactation strategies, ranging from extreme capital to extreme income breeders. Pinnipeds are therefore a good model for understanding females' foraging strategy during lactation.

Weddell seals (*Leptonychotes weddellii*, Lesson 1826) are dependent on the Antarctic sea‐ice during their whole life cycle. The sea‐ice zone not only constitutes the breeding and annual foraging habitat of large populations of ice‐based species, but also a major feeding area for long ranging subantarctic species in winter, pointing at its far‐reaching ecological importance (Hindell et al., 2020). Studying predator–prey relationships is a fundamental approach to reflect internal changes of organisms and ecosystem's status (Spitz et al., 2014; Trathan et al., 2007). Lactation is a critical period of the Weddell seal life cycle (Sapriza, 2019). Females spend from 3 weeks up to 3 months with their pups (Shero et al., 2018). Their energetic constraints are important with drastic changes in energy budget, enhanced vigilance, and pup care (Shero et al., 2018). During the breeding and lactating period, females haul out in groups of 5–100 individuals, accessing water through ice holes that they create and maintain by abrading the ice with their teeth. Anticipating the very high energetic demand from their pup, they accumulate body reserves during gestation. Although most phocid seals are believed to fast during lactation (capital breeding strategy), the length of Weddell seals' lactation period and their high requirement of absolute energetic transfer suggest females must forage during lactation, to cover at least part of their need (Costa, 1991; Foster‐Dyer et al., 2023; Hindell et al., 2020; Sato et al., 2002; Wheatley et al., 2008). The diving behavior of Weddell seals and the link to their foraging ecology has been widely studied after their molt during fall, winter, and spring (Fuiman et al., 2007; Goetz, 2015; Heerah et al., 2013; Schreer & Testa, 1996; Testa, 1994). In summer, during breeding and lactation periods, most fine‐scale analyses of foraging ecology focused on males and non‐lactating females (Davis et al., 2013; Madden et al., 2015; McIntyre et al., 2013; Naito et al., 2010; Watanabe et al., 2003). Studies on lactating females were based on the fluctuation of mothers' weight, collection of biological samples, or on the interpolation of 3D dives tracks using acoustic positioning systems, inferring foraging ecology from dive patterns only (Hindell et al., 2002; Testa et al., 1989; Wheatley et al., 2008). Some studies also gave a fine‐scale description of their swimming behavior, without considering the whole foraging trips (Sato, Mitani, Cameron, et al., 2003).

New perspectives are now offered by miniature animal‐borne sensors. Their technological development allows the recording of predator movements in three dimensions, and they can gather high‐quality data on the physical environment in situ, giving new insight into how these parameters influence their foraging strategies (Heerah et al., 2013; Hussey et al., 2015). Three recent studies of southern elephant seals (*Mirounga leonina*, Linnaeus 1758) have demonstrated the use of animal‐borne sonar tags to estimate fine‐scale predator–prey interactions by assessing the prey field and its characteristics with concurrent predator foraging behavior (Chevallay et al., 2023; Goulet et al., 2019; Tournier et al., 2021). Sonar tags provide the opportunity to study the relationship between foraging behavior of diving animals and their biotic environment, a task that is difficult to implement with video cameras which are highly constrained by light and battery duration. The sonar, that simulates a biological echolocation system, uses high rate active acoustic technology to record information (acoustic density, distance from the sonar, and movement relative to the sonar) on any object in the path of its signal. Such fine‐scale measurement of prey and predator–prey interactions taking place at scales of meters per second cannot be obtained at the relevant spatial and temporal scales with conventional means (e.g., trawling or active acoustic measurements from a research vessel, which operates at scales of 10–1000s of meters and over hours‐days). To the best of our knowledge, available fine‐scale studies dedicated to the quantitative description of the feeding behavior of lactating females did not take into account information on the prey available to seals (Mitani et al., 2003; Sato et al., 2002), although some studies used cameras to identify targeted prey (Foster‐Dyer et al., 2023). Weddell seals are believed to feed on bentho‐pelagic prey, mostly Antarctic silverfish or other notothenioid fish (Burns et al., 1998; Fuiman et al., 2007; Goetz et al., 2017; La Mesa et al., 2022). The use of sonar tags provides additional details on the prey ecology, such as their habitat use and camouflage techniques, giving additional evidence to identify potential prey types.

Sonar tags were deployed on three breeding female Weddell seals in Terre Adélie (East Antarctica) in November 2019, to study animals' movements and dives at high resolution (3D acceleration, magnetometry, time and depth, and GPS location), as well as information on prey and predator–prey interactions using acoustic data (Goulet et al., 2019). These sonars tags also included 3D acceleration and magnetometry sensors providing unprecedented data to document the approach and pursuit of prey by predators. The objective of this study was therefore to assess (i) whether and how lactating female Weddell seals feed (frequency, depth, and duration) during this critical phase of their life cycle, fundamental for understanding their ecology, (ii) what is their utilization of a limited foraging area (benthic or pelagic dives) as they are spatially constrained by the presence of their pup, and (iii) how can we characterize their foraging dives and the approach/catching phases using new tools providing a more detailed description of their behavior. To address our objectives, we combined different approaches based on high‐resolution dive and acoustic data, providing multiple, complimentary views of the females' fine‐scale foraging strategies.

## 2 METHOD

### 2.1 Ethics statement

This work was supported by the French Polar Institute (IPEV) program 1182 ASSET, the “Sentinel of the Sea‐Ice Environment” SENSEI BNP‐Paribas Foundation project, and the CNES‐TOSCA program “Weddell seals bio‐oceanographers of the Antarctic sea‐ice” (P.I.: J.B. Charrassin and S. Labrousse). All experiments were conducted under the ethical regulation approval of the French sub‐Antarctic and Antarctic territories (TAAF) ethic committee. All animals in this study were treated in accordance with the “Comité de l'Environnement Polaire” Ethic Committee guidelines and the work was conducted under permit #2019‐107 of Terre Australes et Antarctiques Française.

### 2.2 Field deployment, animal handling, and devices

**FIGURE 1:**
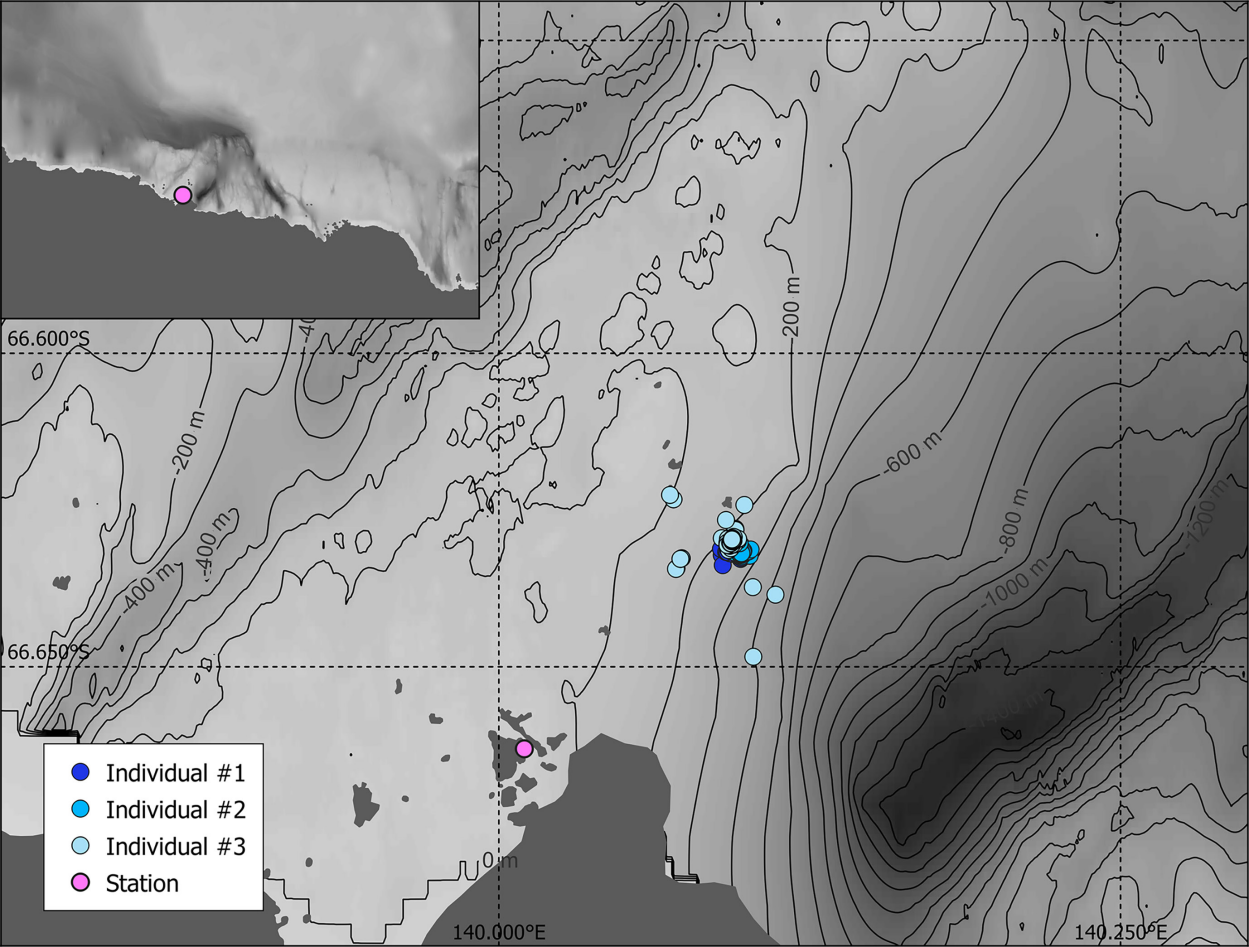
Map of the study area with bathymetry, Dumont d'Urville station location and GPS positions from the three lactating female Weddell seals equipped in November 2019. The inset on the top left shows a broader view of the studied area. Bathymetry data come from Beaman et al. (2011), at a resolution of 100 m.

Three lactating females, hereafter referred to as individual #1, #2, and #3, were equipped with the tags in November 2019 next to the Astrolabe Glacier, near Dumont d'Urville station (66°40′ S, 140°01′ E), in East Antarctica (Figure 1; Appendix S1). Deployment started on November 11th (two individuals) and November 12th (one individual) and ended on November 17th and 18th (7 days deployment), respectively. During this period, the sun was above the horizon almost 24 h/day, although its elevation changed throughout the day. Females were temporarily captured with a canvas head‐bag and anesthetized with a 1:1 combination of Tiletamine and Zolazepam (Zoletil), at a dosage of 0.5 mg/kg (Wheatley et al., 2006a). Seals were monitored until fully recovered from anesthesia and were allowed to go back to sea when no longer sedated. The tags were glued to the fur using quick‐setting two‐part epoxy (Araldite AW 2101 and Hardener HW 2951). Two types of equipment were deployed: (i) an Argos‐transmitting GPS tag was glued on the back of the animal, and recorded GPS position at the surface (Wildlife Computers Inc., Splash10‐BF, 86 × 55 × 27 mm, 132 g in air); (ii) a sonar tag designed by M. Johnson and P. Goulet, containing several types of sensors (detailed below) (SMRU tags, 85 × 44 × 32 mm, 120 g in air) was attached on the head of the animal. In addition, a camera (Little Leonardo, Digital Video logger DVL400M028, 52 mm × 20 mm × 11 m, 15 g in air, 16GB, resolution 1280 × 960) was attached to the side of the sonar, pointing in the same direction. However, video cameras were unable to successfully identify seal prey. The sonar tag recorded pressure and magnetometry at 50 Hz. It also recorded acceleration in the three axes at 250 Hz (individuals #1 and #2) or 200 Hz (individual #3). These data were used to detect prey capture attempts and to reconstruct fine‐scale movements and orientation of the seal during dives (detailed in sections 2.3 and 2.4) (Figure 3a). The tag was configured to emit 10 μs pings at a rate of 25 Hz (i.e., 25 sound signals per s), with a ‐3 dB beamwidth of approximately 3.4° and detection range of 7 m. The tag was programmed to record continuously throughout the deployment. For one individual, the depth sensor stopped working properly after approximately 142.3 h (the 370th dive, at the end of the fifth day) until the end of the deployment. Acoustic data provide a fine‐scale description of the interaction between the seal and prey. It also provides the opportunity to precisely detect benthic dives, when the ocean floor was visible on echograms (i.e., images of acoustic data).

### 2.3 Movement data analyses

Data analysis was conducted in Matlab version R2021b (The MathWorks, 2014), using functions from www.animaltags.org (unless stated otherwise) (Animal Tag Tools Wiki, 2017), and in R Studio software, version 1.3.1093 (R Core Team, 2020). Movement data (depth, magnetometry, and triaxial acceleration) were calibrated using custom‐made script and function *do.cal* (Animal Tag Tools Wiki, 2017) (Figure 3b). Magnetometry was calibrated based on the location of true magnetic south and date. Magnetometry and depth were downsampled to a sampling frequency of 5 Hz for the rest of the analysis. Acceleration data were kept at their full bandwidth (200 or 250 Hz, depending on the individual). Magnetometry, depth, and triaxial acceleration data were used to infer seal's orientation during their dives and their diving track (Figure 3b). Dive profiles were assessed using depth data. Dive data followed a bimodal distribution, therefore two types of underwater activities were identified with a depth threshold of 5 m (no drift in the tag measure was observed, the surface remained at 0 m throughout the deployment): (i) underwater activity between 0 and 5 m (depth measured using the pressure sensor, dive data >0 m were excluded), and (ii) dives deeper than 5 m (Appendix S2). The sun angle was computed, using the function *SolarAzEl* (Animal Tag Tools Wiki, 2017), and the period of the day was assessed following Kirkwood and Robertson (1997) and Labrousse et al. (2019) (Figure 3c). Daytime was defined as a solar angle ranging from 0° to +∞°, twilight between −12° and 0°, and night between −12° and −∞°.

### 2.4 Prey capture attempt detection

**FIGURE 2:**
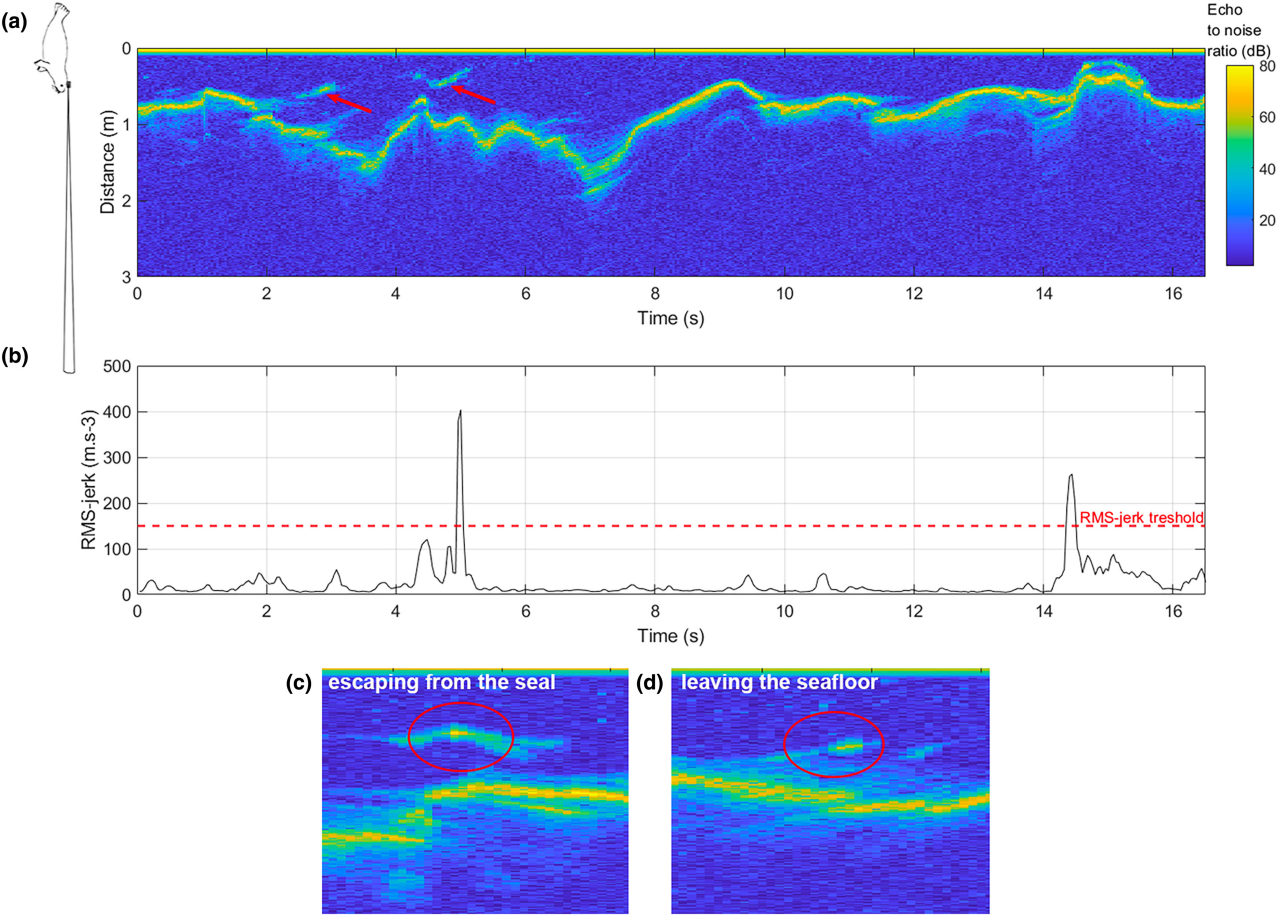
Example of an echogram (a) with associated RMS‐jerks (b), in a 16‐s time window. The horizontal axis represents time in seconds, and the vertical axis is the sonar range, that is, the distance between target and the sonar tag, in meters, with the sonar at 0 m. The seal sketch on the left of panel (a) indicates the position of the animal during recording and the sonar beam orientation (Chevallay et al., 2023). The colors represent the echo‐to‐noise ratio (ENR), resulting from the subtraction of the background noise in decibels, to only highlight the echo signal (Goulet et al., 2019). The ENR can be interpreted as the density of objects that reflect the sonar signal. In panel (a), red circles indicate the position of a potential prey. The line around 1 m distance is the seafloor. The red line in panel (b) indicates the threshold at which a RMS‐jerk peak is identified as a PrCA. The two other panels show the prey reactions, either escaping from the seal (panel c) or leaving the seafloor (panel d).

**FIGURE 3:**
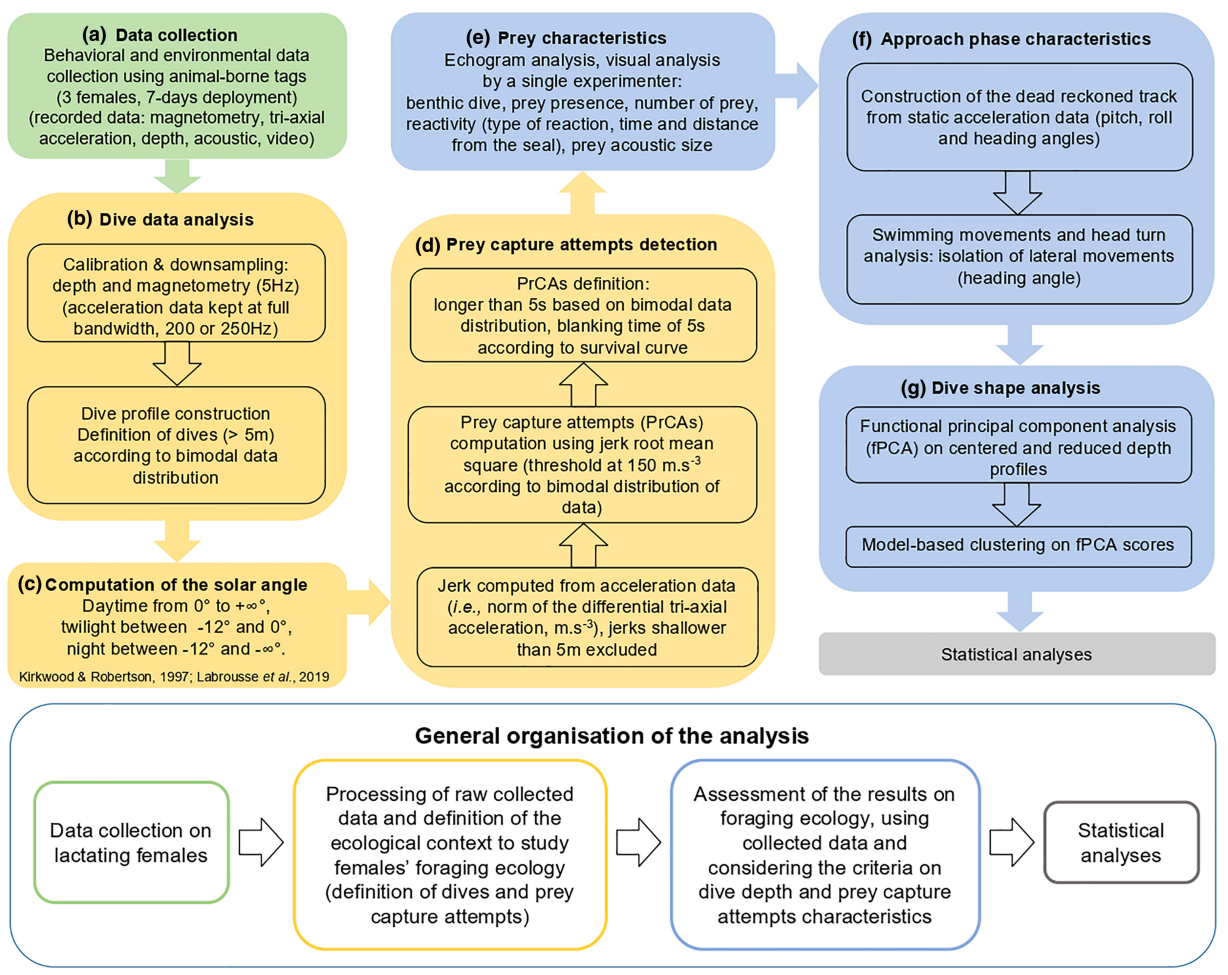
Workflow of the data analysis preceding the statistical analysis of the results. This figure illustrates the analysis pathway, from collecting (green box) and processing raw data (yellow boxes) to extracting ecological results (blue boxes), that allowed us to give a fine‐scale description of Weddell seals' foraging ecology during lactation.

Weddell seals are categorized as biting feeders due to their craniofacial musculature (Davis et al., 1999; Kienle et al., 2022). They capture their prey by opening their mouth and quickly closing jaws on the prey (Naito et al., 2010). Prey capture attempts (hereafter PrCAs) were therefore detected using rapid acceleration in the three axes, accounting for head movements since the tag was placed on the head of the animal (Naito et al., 2010). To detect these movements, the jerk (i.e., the norm of the differential three‐axial acceleration, in m/s^3^, Ydesen et al., 2014) was calculated (Figure 3d). The jerk root mean square was used to provide an estimation of the jerk magnitude (Figure 3d). Root mean squares of the jerk above 150 m/s^3^ were classified as PrCAs, according to bimodal distribution of data (Appendix S3). This threshold was used to determine the start and end times of PrCAs: A PrCA starts when the jerk exceeds the threshold, and ends when the jerk drops below the threshold. Events shorter than 5 s were ignored, based on data distribution, and on the fact that the facial musculature of Weddell seals implies that the capture must last at least for a few seconds (Figure 3d). To distinguish between a long PrCA (composed of several peaks) and single successive PrCAs (composed of a single peak), a survival curve was computed to establish the smallest time interval between two PrCAs events (Goulet et al., 2020). This time threshold was set here at 5 s (Figures 2 and 3d). The resulting PrCAs were compared to processed sonar data, to check whether a prey item was visible in sonar data. Only prey capture attempts occurring during dives below 5 m were studied as sonar data were difficult to use in the under‐ice area.

### 2.5 Sonar data

#### 2.5.1 Processing of sonar data

Sonar data analysis was performed in Matlab version R2021b (The MathWorks, 2014), using functions from www.animaltags.org (unless stated otherwise) (Animal Tag Tools Wiki, 2017).

Sonar data were analyzed as echograms (Figure 2a). Echograms display echo signals recorded by the sonar, showing the time on the horizontal axis and distance from the sonar on the vertical axis. A color scale is set to represent the intensity of the signal (Figure 2a). Each vertical line corresponds to a ping, that is, a sound emission. As the seal moves underwater, the sonar emits ultrasounds that are reflected off particles or objects contained in the acoustic beam (Goulet et al., 2019). Echograms display the object distance from the sonar as a function of time (Figure 2a); therefore, an object getting closer to the sonar would be represented as a downward trace, and vice versa regarding an object moving away from the sonar (Goulet et al., 2019).

#### 2.5.2 Analysis of prey characteristics

Analyses were performed on echograms within 5 s before and after prey capture attempts identified from acceleration data (Figure 3e). The root mean square (RMS) of jerk data was displayed under the corresponding echogram. To balance potential bias induced by visualization of the RMS‐jerk, all echograms with PrCAs were analyzed randomly. Several variables were extracted from a visual inspection of each echogram by a single examiner, and used as descriptors of the predator–prey interaction (Figure 3e) (Goulet et al., 2019): (i) Presence of the seafloor on the echogram to infer whether the hunting phase was benthic or pelagic; (ii) Presence of a clear prey trace (Figure 2a); (iii) Number of prey, either “one” or “several,” to distinguish between single prey echoes or more than one prey echo within the same ping (i.e., within the same vertical line), accounting for multiple prey (Chevallay et al., 2023; Goulet et al., 2019; Jones et al., 2008); and (iv) Prey reactivity: whether the prey reacted to the presence of the seal, and if so, the type of reaction (Figure 2c,d) (Chevallay et al., 2023). Two types of reaction were distinguished: “escaping from predators,” when the prey was just above the seafloor and swam away from the seal (Figure 2c), and “leaving the seafloor,” when the prey was hidden within the seafloor and left its hideout as the seal approached (Figure 2d). To distinguish between the two types of prey reaction, we observed the shape of the prey signal on the echogram: A straight line with negative slope and departing from the seafloor signal was classified as “leaving the seafloor,” and a curved line far from the seafloor with negative and then positive slope was classified as “escaping.” When no reaction could be distinguished, the prey was classified as “not reacting.” The time after the start of the PrCA and the distance from the sonar at which a reaction was detected were also extracted; (v) Prey acoustic size (Appendix S4), estimated via the number of pixels within prey trace in a single ping (i.e., single column of pixels) (Goulet et al., 2019). The prey size was only measured for reactive prey images that were clearly separated from other images on the echogram. The acoustic size must be distinguished from the actual prey size, as it only represents the size of the portion of prey that was in the path of the sonar beam. However, it is a useful tool to compare relative size difference among prey since bigger prey should appear larger in acoustic data.

### 2.6 Behavioral and orientation data

#### 2.6.1 Data processing

For each PrCA where a prey was detected on the echogram, we studied the behavior of the seal while approaching the prey: Seal's orientation and swimming behavior were extracted using magnetometry and lateral acceleration (Figure 2f). The approach phase was defined as the 10 s preceding prey capture attempts. Accelerometers recorded both the dynamic acceleration related to the movement of the seal, and the static acceleration, which corresponds to the orientation of the device with respect to the Earth's gravitational field (Shepard et al., 2008). Pitch (rotation around the lateral axis), roll (rotation around the antero‐posterior axis), and heading (rotation around the dorso‐ventral axis) angles were calculated (Figure 2f), from accelerometer and magnetometer data. The seal 3D movement before each PrCA was created by dead‐reckoning the track: This method is a 3D reconstruction of the animal's trajectory, in local coordinates (Figure 3f) and requires speed and direction of the animal between every GPS position (Laplanche et al., 2015). Pitch, roll, and heading speed and angle must also be computed. The function *depth_rate* (Animal Tag Tools Wiki, 2017) was used to compute the mean vertical speed and the dead‐reckoned track was retraced using the function *ptrack*.

#### 2.6.2 Swimming and diving behavior

Lateral acceleration was filtered to reduce noise and isolate swimming movements (Figure 3f). Nonfiltered acceleration patterns desynchronized with filtered lateral movements were attributed to other types of lateral movements, such as head turns (Figure 3f). We characterized the seals' behavior when approaching a prey item, by extracting several parameters over a time window from 10 s before to 5 s after the end of the PrCA. We visually assessed whether (i) the seals were chasing prey before or after the recorded PrCA; (ii) the orientation of the seal (its pitch, roll, and heading angle); (iii) its swimming movements (number of peaks in the filtered lateral acceleration data); and (iv) other lateral movements before the start of the PrCA when prey reaction was observed, at the time of the reaction.

### 2.7 Statistical analysis

Statistical analysis was conducted with R Studio software, version 1.3.1093 (R Core Team, 2020). Former studies on the foraging ecology and diving pattern of marine predators have demonstrated that the shape of the dive profile is an important indicator of the type of activity performed during dives (Davis et al., 2003; Godard et al., 2020; Schreer & Testa, 1996). Applying this analysis to foraging behavior gives an insight into the structure of such dives, and allows us to assess whether the seal swims directly to the bottom to hunt or displays a more sinuous descent, if such dives are composed of several ascents and descents or a single diving phase, etc. Dive shape analysis is a tool to characterize the behavioral specificity of foraging dives and the typical patterns that distinguish them from other dives. We built clusters of dives shapes to allow for identification of stereotyped patterns, that can be used as functional indicators of different types of activities. Following Godard's work, functional principal component analysis (fPCA) was applied to high‐resolution dive data, centered and reduced on depth and time (Godard et al., 2020). The package *fda* was used (Ramsay et al., 2021). This first reduction step allows for comparison among dive profiles, reducing bias induced by the different number of sampling points per dives, distance between sample points and scale. A second reduction is performed through fPCA: Reduced dive curves are projected into a small dimension space, using principal component scores that reflect deformation of the curve. These scores are used to perform clustering to identify dive shape characteristics. Prey characteristics and approach behavior were then compared between resulting dive shape clusters. Three types of clustering methods were tested on fPCA scores: model‐based clustering (package *mclust*, Scrucca et al., 2016), K‐means method, and hierarchical clustering (using Ward's method) (package *cluster* version 2.1.0, Maechler et al., 2019) (package *factoextra* version 1.0.7, Kassambara & Mundt, 2020). Their performances were compared using pairwise comparison for corrected‐for‐chance rand index (c‐RI) and variation of information index (VI), using function *cluster.stats* on distance matrices (Appendix S5). Using these indexes and following Godard et al. (2020), the model‐based clustering method was selected. Different thresholds of probability for a given dive to be associated with a given cluster were tested. Looking at the number of resulting dives for every tested threshold, dives classified with a probability of 80% were kept. Differences between individuals (explanatory parameters) were assessed for the presence of prey and benthic/pelagic dives (in order to explain differences in prey presence or pelagic dives in function of interindividual differences) using generalized linear models following Bernoulli distribution. Only two seals out of three had prey presence on echograms, therefore an ANOVA was performed to compare prey reaction, size, distance, and reaction time between the two individuals. The effect of the time of the day (twilight and daytime; explanatory parameters) on each dive characteristic (dive duration, depth; in order to explain dive characteristics in function of the time of the day) was also tested using general linear model with a Bernoulli distribution. Using linear regression models, prey characteristics (prey size, reaction time, and distance from the seal) were modeled as a function of the solar angle (providing information on the time of day, twilight, and daytime) (in order to explain prey characteristics in function of the solar angle).

## 3 RESULTS

### 3.1 General diving behavior

**FIGURE 4:**
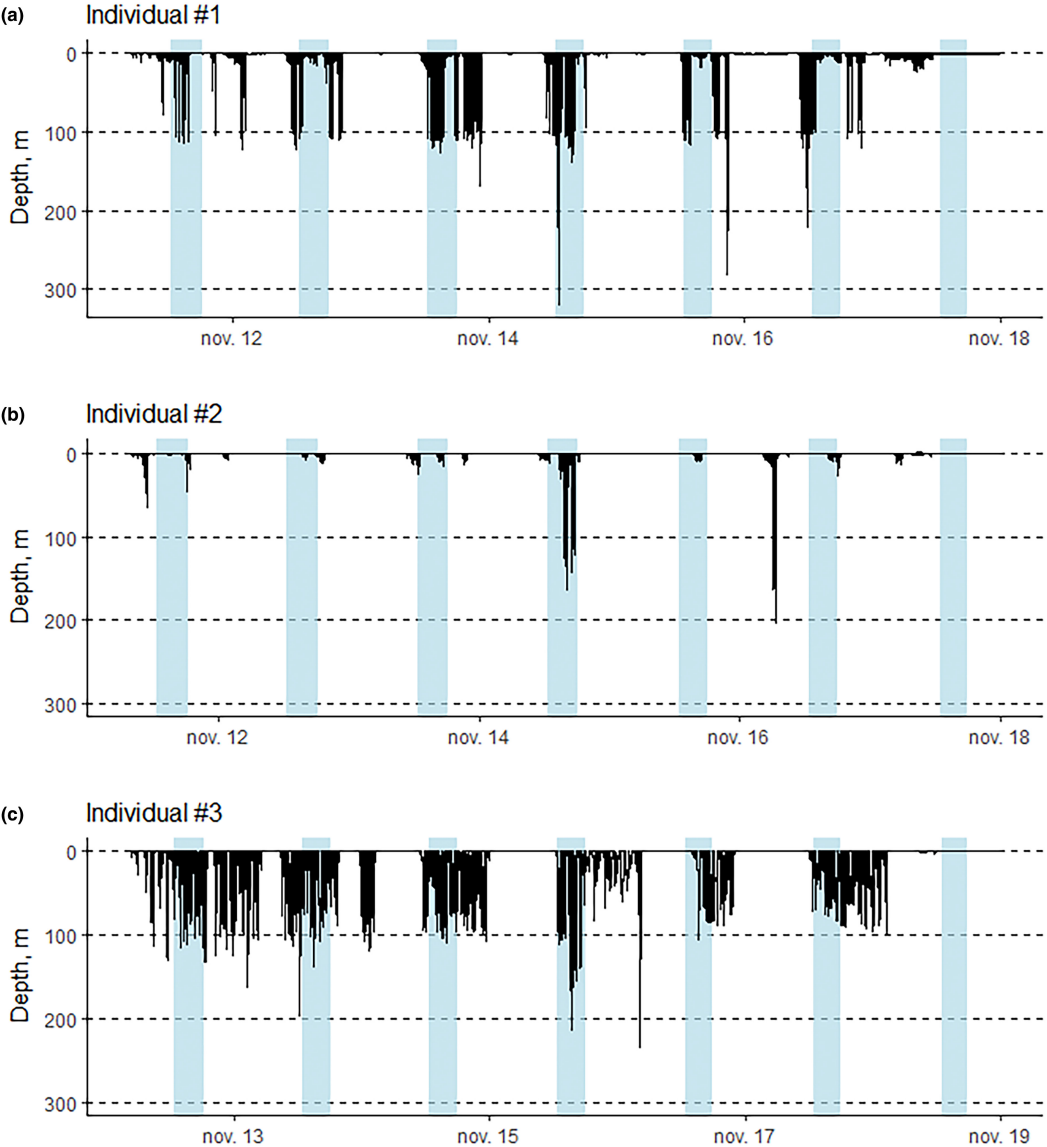
Dive profiles of the three studied female Weddell seals during the 7 days of deployment, with individual #1 in panel (a), individual #2 in panel (b), and individual #3 in panel (c). Panel (a) is the dive data of the individual whose data logger stopped working properly toward the end of the deployment. Blue shading indicates twilight period.

A total of 854 dives were recorded among all three individuals (Table 1, Figure 4). Most of the time spent underwater was dedicated to diving: seals spent 4.5 more times diving than swimming at shallow depth (<5 m) (Table 1).

**TABLE 1:** Summary of the tracking and video data recorded by the three female Weddell seals equipped in 2019 during lactation in Terre Adélie.

		Individual #1	Individual #2	Individual #3	Total
Microsonar data					
Dive depth, m (deep dives only, >5 m)	Total number of dives	369	165	320	854
	Time spent at shallow depth, h (<5 m)	10.6	6.8	4.3	21.7
	Time spent at deep depth, h (>5 m)	28.8	7.2	62.5	98.5
	Maximum	320	203	234	320
	Minimum	5	5	5	5
	Mean ± SD	34 ± 46	16 ± 30	48 ± 42	36 ± 44
Dive duration, min (deep dives only, >5 m)	Maximum	18	13	55	55
	Minimum	> 0.1	0.1	> 0.1	> 0.1
	Mean ± SD	3 ± 4	3 ± 2	11 ± 10	7 ± 8
GPS data	Mean number of GPS points per day	36	36	29	29
	Mean distance between GPS points (km) ± SD	0.2 ± 0.1	0.2 ± 0.1	0.3 ± 0.3	0.2 ± 0.2
	Mean maximum radius of track in 1 day (km) ± SD	1 ± 2	3 ± 2	2 ± 2	2 ± 2
Acceleration data	Number of PrCAs, >5 s	308	2	21	331
	Number of dives with PrCAs	61	2	15	78 (9.13%)
	Mean number of PrCA per dive ± SD	5 ± 4	1	1 ± 1	4 ± 4
	Mean depth of PrCAs, m	92 ± 27	85 ± 51	29 ± 21	88 ± 30
	Number of benthic dives, identified by acoustic	301	2	20	323 (98%)
	Number of PrCAs with prey traces	116	0	9	125 (38%)
Video data					
	Recording, h	2	1.5	2.3	5.8
Pup interaction	Unused images	3%	14%	65%	32%
	Total time spent with pup	61%	40%	6%	33%
	On sea ice, % of pup interaction	61%	85%	100%	71%
	In water, % of pup interaction	39%	15%	/	29%
Haul‐out	Total time spent in haul‐out	86%	24%	17%	42%
	Not moving, % of haul‐out time	98%	30%	28%	77%
	Moving on sea ice, % of haul‐out time	2%	70%	72%	23%
Underwater	Total time spent underwater	30%	77%	8%	14%
	At the surface, % of underwater time	81%	97%	100%	92%
	Diving, % of underwater time	19%	3%	/	8%
	Time spent maintaining ice holes	15%	20%	/	10%
	Time spent interacting with other seals	/	3%	1%	1%

Abbreviation: SD, standard deviation.

On average, dives lasted for 7 ± 8 min and reached a mean depth of 35 ± 44 m (Table 1). Two individuals had similar mean dive duration (3 ± 4 min, and 3 ± 2 min, respectively) compared with the third one (11 ± 10 min). One individual performed much shallower dives than the two others on average (16 ± 30 m, as opposed to 34 ± 46 and 48 ± 42 m, respectively), with no apparent link between mean dive depth and mean dive duration differences among individuals (Table 1). Dive depth (maximum 320 m, 203 m, and 234 m for the three individuals, Table 1) was affected by solar angle: daytime dives were significantly deeper than twilight dives (ANOVA, *p* < .001, *F*_1, 1169_ = 76.94, and adjusted *R* ^2^ = .06095). The sun angle varied between −5.89 and 42.27: during this period of the year, there is no night and twilight lasts for about 4 h. Most dives happened during the day (77%, Table 1). The total number of PrCAs recorded is 331 among all individuals (Table 1). From the GPS data, the computed mean distance traveled among seals each day within a radius (i.e., the maximum distance from the first recorded position of the day) was 5 ± 2 km (Table 1). Individuals distributed evenly in all directions near the deployment location and showed no orientation preferences or patchy distribution (Figure 1b). A total of 6 h of video footage were analyzed, among which 32% of images were unusable (Table 1) due to presence of ice obstructing the view, twitches due to rapid movements of the seal or luminosity being too low. Differences were observed among individuals with respect to the type of activities performed and time dedicated to each activity. The age of the pup is unknown for all three individuals; therefore, a potential relationship between activity partitioning and pup age cannot be investigated. One female (individual #1) spent most of the time hauled‐out (86% of overall recorded time), whereas another female spent most of its time in the water (77% of time), remaining at the surface (depth < 5 m) and plunging its head at times (Table 1). Interaction with pup was the second most important activity for two individuals, mostly on sea‐ice (37% and 34% of overall recorded time, respectively). The seals were also recorded abrading ice with their teeth to maintain ice holes (Table 1).

### 3.2 Benthic dives

The ocean floor was visible on studied echograms most of the time (98% of total echograms, Table 1). PrCAs from these echograms and corresponding dives were therefore categorized as benthic. Benthic dives represent 95%–100% of dives for each individual (Table 1). There were fewer benthic dives as sun angle got lower (twilight) (67% of benthic dives during the day, 33% at twilight), but this difference was not statistically significant (GLM, *p* = .588, *z* = −0.542). The water column was divided in depth bins of 40 m. More benthic dives took place in the 80–120 m depth bin than in any other 40 m range from the surface to the deepest dive (GLM, *p* = .0140, *z* = 2.458).

### 3.3 Dive shape

**FIGURE 5:**
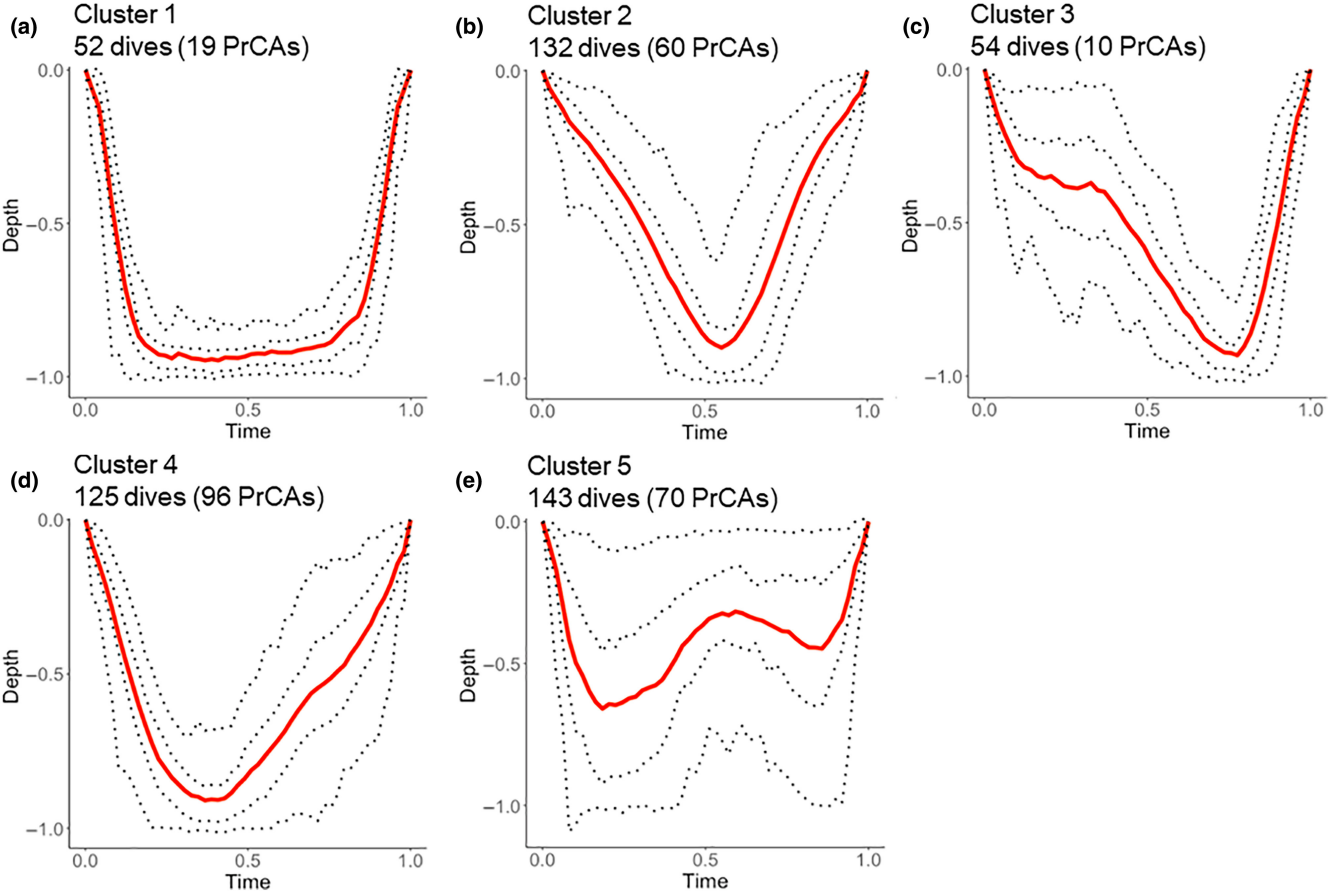
Shape of the different dive clusters (dives were attributed to each cluster with a certainty of 80%). Average cluster curves are in red. Dotted lines are the 2.5%, 25%, 75%, and 97.5% quantile curves. (a): cluster 1, (b): cluster 2, (c): cluster 3, (d): cluster 4, and (e): cluster 5.

The first three dimensions of the functional principal component analysis that account for most of the variance were retained. All three dimensions were related to sinuosity: whether the sinuosity at the end of the dive was higher than the one at the beginning (accounting for 33.4% of the variance), whether the dives demonstrated any sinuosity at all at the beginning or at the end of the dive (24.5% of the variance), and whether the highest sinuosity was at the beginning/end of the dive or at the bottom (middle) of the dive (20.5% of the variance). We then computed model‐based clustering on the three principal components, and we set a threshold of 80% certainty for a dive to belong to a given cluster. Final clusters covered 506 of the 854 dives and each cluster contained PrCAs. Of the 331 recorded PrCAs, 255 were classified into dive clusters and the PrCAs were inequitably assigned between the clusters (Table 2). Very few PrCAs were recorded in cluster 3 (Table 2, Figure 5c) and a few more in cluster 1 (Table 2, Figure 5a), whereas the majority of PrCAs occurred during dives in clusters 2 (Figure 5b, 60 PrCAs), 4 (Figure 5e, 96 PrCAs), and 5 (Figure 5d, 70 PrCAs) (Table 2). Cluster 1 shapes (Figure 5a) were characterized by a direct ascent and descent phase, with a longer bottom phase than other clusters. The space between quantiles represents the sinuosity of the dive, that is, the straightness of track. Cluster 2 dives (Figure 5b) had a V shape without skewness and vertical sinuosity during the whole dive. Cluster 4 dives had a V shape and were skewed on the right (Figure 5d). Therefore, they had a rather straight descent and a higher vertical sinuosity during the ascent phase (dive curve quantiles were more spaced on the right than on the left), as opposed to cluster 3 that was left‐skewed with a rather straight ascent (Figure 5c). Dives in cluster 5 were much shallower than other dives and displayed a W shape (Figure 5e).

**TABLE 2:** Number of dives per cluster, and number of PrCAs per cluster for each individual and in total (for a given certainty of 80%).

	Total	Cluster 1	Cluster 2	Cluster 3	Cluster 4	Cluster 5
Number of dives						
Individual #1	193	15	61	17	47	53
Individual #2	114	21	20	7	22	44
Individual #3	199	16	51	30	56	46
All individuals	506	52	132	54	125	143
Number of PrCAs						
Individual #1	239	15	57	10	87	70
Individual #2	1	0	0	0	1	0
Individual #3	15	4	3	0	8	0
All individuals	255	19	60	10	96	70

### 3.4 Prey characteristics

For one individual (individual #2), no prey was detected on the echograms. This individual was therefore excluded from analyses on prey characteristics. Regarding prey number, two prey images were categorized as schools of prey (one for each individual). The rest were categorized as single prey. Fifty six percent of prey were reactive (70 reactive prey out of 125, Table 3). We were able to classify every prey reaction within the two reaction categories (i.e., escaping or leaving the seafloor). Prey leaving the seafloor were observed in 70% of cases (49 out of the 70 reactive prey, Table 3). Prey reaction was not significantly affected by solar angle (GLM, *p* = .936, z = 0.080) and depth (GLM, *p* = .634, z = 0.476). PrCAs occurred at a depth of 88 m on average, with a mean of 4 PrCAs per dive and nearly all PrCAs were benthic (98% of PrCAs) (Table 1). There was a significant difference in prey acoustic size between the two individuals (ANOVA, *p* < .001, *F*_1, 94_ = 11.99, and adjusted *R* ^2^ = .103), with a mean ± standard deviation of 5 ± 1 cm and 6 ± 3, respectively (Table 3). There was also a significant difference among individuals in prey reaction distance from the sonar (ANOVA, *p* = .027, *F*_1, 68_ = 5.115, and adjusted *R* ^2^ = .056), with a mean of 50 ± 17 cm and 68 ± 17 cm, respectively (Table 3). On the contrary, there was no significant difference among individuals regarding prey reaction time after the start of the PrCA (ANOVA, *p* = .314, *F*_1, 68_ = 1.029, and adjusted *R* ^2^ = .0004) (mean time of 9 ± 7 s). The maximum recorded distance at which a prey reacted to the seal's approach was 121 cm (Table 3).

**TABLE 3:** Summary of PrCAs' echogram analysis regarding prey characteristics, for the two studied Weddell seal females.

	Individual #1	Individual #2	Total
Number of preys			
Number of PrCAs with schooling prey traces	1	1	2
Number of PrCAs with single prey traces	115	8	123
Prey reaction type			
Number of PrCAs with reacting prey traces	65	5	70
Number of PrCAs with traces of preys leaving the bottom (reaction A)	45	4	49
Number of PrCAs with traces of preys escaping (reaction B)	20	1	21
Number of PrCAs where the reaction could not be distinguished	0	0	0
Prey reaction distance from the sonar, cm			
Maximum	121	97	121
Minimum	16	55	16
Mean ± SD	50 ± 17	68 ± 17	51 ± 18
Prey reaction start time after beginning of recorded PrCA, s			
Maximum	43	11	43
Minimum	>0.1	>0.1	>0.1
Mean ± SD	9 ± 7	6 ± 5	9 ± 7
Prey size, cm			
Maximum	10	13	13
Minimum	3	5	3
Mean ± SD	5 ± 1	6 ± 3	5 ± 1

Abbreviation: SD, standard deviation.

There was no significant difference in prey reaction distance from the seal according to the time of the day (daytime or twilight) (ANOVA, *p* = .098, *F*_1, 68_ = 2.82, and adjusted *R* ^2^ = .026). Similarly, prey reaction time after the start of the PrCA did not significantly change according to the time of the day (ANOVA, *p* = .6, *F*_1, 68_ = 0.278, and adjusted *R* ^2^ = −.01), and depth (ANOVA, *p* = .744, *F*_1, 68_ = 0.107, and adjusted *R* ^2^ = −.01). However, prey reaction distance increased with depth (ANOVA, *p* = .029, *F*_1, 68_ = 4.965, and adjusted *R* ^2^ = .054).

### 3.5 Approach behavior

**FIGURE 6:**
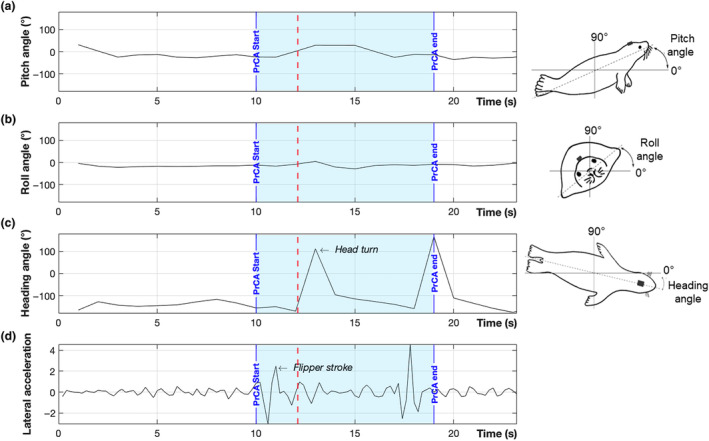
Example of pitch angle (a), roll angle (b), heading angle (c), and lateral acceleration (i.e., swimming movements) (d) curves 10 s before, 5 s after and during a PrCA, for one individual female Weddell seal (individual #1). The blue section is the duration of the PrCA. The red dotted line indicates the moment when the prey reacted, according to the corresponding echogram. While pitch (a) and roll (b) angles remain close to 0°, a rapid change (less than 2 s) in heading angle is visible (c), concurrent with prey reaction. The seal demonstrates no rapid swimming movement before the start of the PrCA (d), but a flipper stroke is visible just after the start of the PrCA, before prey reaction (d). Figures of seals are on the right side of panels (a), (b), and (c) to illustrate the seal's orientation (Chevallay, 2020).

**FIGURE 7:**
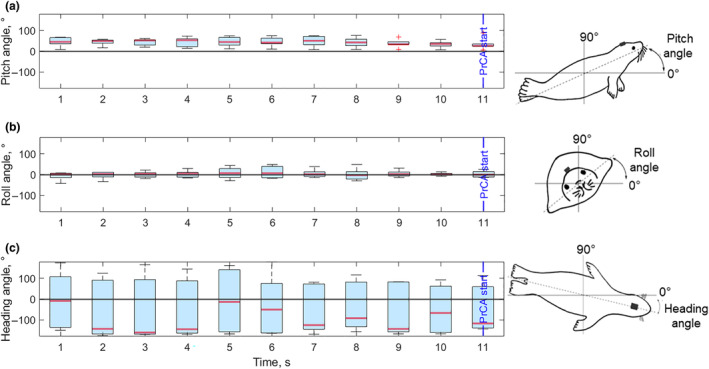
Example of pitch (a), roll (b), and heading (c) angle boxplots, second per second for every PrCA belonging to cluster 4, for one female Weddell seal. The time on the horizontal axis includes the approach phase (1–10 s), the 11th second (blue solid line) being the start of the PrCA. The black line indicates 0° for the three body angles. The oscillations in median value (red lines) in panel (c) illustrate alteration in heading angle throughout the approach phase, as opposed to almost static pitch and roll angles. Figures of seals (Chevallay, 2020) are on the right side of each panel to illustrate seal's orientation.

We only analyzed the approach behavior of individuals (*n* = 2) for which reacting prey were visible on the echograms. For most PrCAs, the seal was actively swimming 5 s before the prey reacted (74%, Table 4), and kept swimming during the 5 s following prey reaction (79%, Table 4). Such behavior was visible on filtered lateral acceleration data (Figure 7d). All individuals displayed a periodic alternation of heading angle, from negative to positive values, throughout the approach, that is, turning their head from one side to another (Figure 6c). Using a large time window around PrCAs, from 10 s before to 5 s after the PrCA, it appeared that most prey reacted during the PrCA (59% of reacting prey), of which 34% of preys reacted before the PrCAs started (Table 4). A minority of prey reacted after the end of the PrCA event (7%). Once the prey reacted, the seals changed their heading angle (70% of the time, ±1 s around time of prey reaction, Table 4) (Figure 7c), turning its head to the left (negative angle) (43% vs 27% to the right, Table 4).

**TABLE 4:** Summary of the seals' approach behavior to reacting prey, from 10 s before to 5 s after the PrCA.

	Number of PrCAs	Reaction time			Flipper strokes		Head turn	
		Before the start of the PrCA (%)	During the PrCA (%)	After the end of the PrCA (%)	Before the start of the PrCA (%)	Continuing during the PrCA (%)	Left turn (negative angle) (%)	Right turn (positive angle) (%)
Individual #1								
Escaping prey	20	20	65	15	80	90	50	25
Leaving the seafloor prey	45	40	55	5	69	71	38	22
Individual #2								
Escaping prey	1	0	100	0	100	100	0	100
Leaving the seafloor prey	4	50	50	0	100	100	75	75
Total								
Escaping prey	21	19	67	14	81	90	48	29
Leaving the seafloor prey	49	41	55	4	71	73	41	27
Total	70	34	59	7	74	79	43	27

There was no apparent difference in approach behavior among dive clusters within the 10 s preceding the start of the PrCA.

## 4 DISCUSSION

This study is a fine‐scale description of the foraging behavior of three female Weddell seals during lactation and the first deployment of sonar tags on Weddell seals. It sheds light on previously unexplored aspects of their foraging behavior prey fields and fine‐scale predator–prey interactions using a combination of different methods and analyses (acceleration data to estimate PrCA events, acoustic data to describe the predator–prey interaction at fine scale and functional PrCA to depict the structure of the foraging dives). This work provides additional evidence that Weddell seal females still forage during lactation, displaying a mix of capital‐breeding and income‐breeding strategies during this period of physiological stress. Females used a restricted foraging area (1–3 km) diving under the sea ice and returning regularly to their breathing holes. Females spent most of their time hauling‐out (77%), 19% of the time was dedicated to diving (>5 m) (854 dives), and 4% to shallower under‐ice activities (<5 m). Most of the dives were benthic dives (97%). A total of 331 PrCAs were identified from triaxial acceleration data and occurred within 78 dives, including 4 ± 4 PrCAs/dive, of which 125 prey were identified from the echograms. All PrCAs occurred on the seafloor, at shallower depth than usual Weddell seal records (88 ± 30 m among all studied individuals).

During the approach phase, seals constantly scanned the area, moving their head left to right suggesting visual prey searching and opportunistic foraging. Most of the prey detected by the sonars were probably hidden in the benthic macrofauna and left the seafloor as the seals approached. It must be underlined that the sample size used here is not sufficient to detect stereotyped behavior and infer any preference in head turning, such as handedness (Friedlaender et al., 2017).

This study suggests that female Weddell seals still forage while lactating, hunting their prey by chasing them from the seafloor. It opens perspectives for more detailed study and increased sample size to assess the position of Weddell seals on the capital breeding/income breeding spectrum.

### 4.1 Foraging behavior from acoustic and accelerometry data

#### 4.1.1 Prey detection

The sonars used in our study have proven to be an efficient tool to analyze the prey field in front of the seals, allowing for accurate characterization of (i) the prey's response to the predator's approach (distance and type of reaction), (ii) its acoustic size, and (iii) ecology of prey (e.g., single benthic fish, remaining close to the ocean floor). Most prey were characterized as single prey, which is in line with the research of La Mesa et al. (2022) conducted in another region of the Antarctic shelf. Using a photographic survey of the ocean floor at Adélie cove (on the coast of Terra Nova Bay, Victoria land), they demonstrated that most fish were single individuals, mostly belonging to the notothenioid taxa (La Mesa et al., 2022). Unfortunately, the cameras used in this study did not prove satisfactory in identifying Weddell seals' prey, since they hunt at depth where the light was insufficient, and no bioluminescent prey were recorded. Similarly, precise prey identification at the taxon or species level using sonar remains limited. Indeed, prey acoustic size is not sufficient to assess the taxon encountered, and supplementary analysis are needed such as stable carbon isotope, stomach content, or feces analysis.

#### 4.1.2 Benthic dive identification using the sonars

Sonar tags present several advantages for assessing the environment surrounding the seal and the seal behavior. For example, they provide information on the presence of the seafloor within the sonar range, allowing for the identification of the type of diving behavior according to these parameters (i.e., pelagic, therefore in the water column under the sea ice, or benthic, close to the seafloor). Former work assessed whether dives were benthic by comparing ocean floor topography with maximum diving depth recorded (e.g., Hindell et al., 2002). However, bathymetry data in Antarctic regions is rather sparse and often at low resolution. Using echograms which display echoes from the seafloor, information on the type of dive (i.e., benthic vs pelagic) was accurate and we demonstrated that most PrCAs took place at ca. 50 cm from the seafloor. The limitation of this method is that if the seal does not point the sonar toward the seafloor, no signal can be detected on the echogram and the dive could falsely be classified as pelagic.

#### 4.1.3 Predator–prey interactions

Using sonar tags to record echograms along with prey capture attempts (PrCAs) provides new insights on fine‐scale foraging strategies and predator–prey interactions. In this study, PrCAs were defined using the acceleration signal, that is, RMS‐jerk (root mean square of the jerk), and the PrCAs identified were compared with echograms. Acceleration data alone may not be sufficient to detect every phase of Weddell seals' PrCAs. In this study, we observed that some PrCAs seemed longer than what the RMS‐jerks detected. Some prey were visible on echograms and reacted to the seal's approach before the recorded start of the PrCA, as detected through acceleration data. In other words, the combination of echograms and acceleration data provides a more complete picture of foraging events and predator–prey interactions. However, it remains impossible to estimate feeding success and determine whether the prey was caught by the seal or not. Further analyses are required to assess costs and benefits of such foraging activities (energetic cost of reducing the number of foraging trips while lactating, reproductive cost of leaving the pup alone on the ice during dives, benefit in terms of survival of not fasting during lactation, etc.), that is, to estimate the energy budget of lactating females, or to assess foraging success. These analyses could use hydrophones, that record swallow and breathing sounds or video cameras with light (Foster‐Dyer et al., 2023). For example, Foster‐Dyer et al. also combined information on the prey species, obtained through video cameras, and the seals' demographic descriptors (breeding history and pup age) to examine foraging behavior (Foster‐Dyer et al., 2023).

### 4.2 Foraging behavior and lactation constraints

Females remained close to their deployment location for the 7‐day recording. The foraging distances observed in our study are consistent with former studies on the fine‐scale spatial use of lactating females demonstrating very restricted foraging area (Larue et al., 2019). Presence of pups may limit females' foraging trip distance, as observed in other marine species such as little penguin (Sutton & Arnould, 2022).

#### 4.2.1 Dive shape analysis

Clustering analysis based on the shape of dives facilitates the identification of the type of activity performed during dives (Davis et al., 2003; Godard et al., 2020; Schreer & Testa, 1996). It provides an insight into the structure of dives, distinguishing diving patterns among all the dive data. These patterns (sinuosity, straightness of the track, bottom time duration relatively to the whole dive, etc.) serve as indicators of the activities performed during the dive (Heerah et al., 2019).

We found that the seals' dives could be partitioned in five clusters, according to their shape, however, these results might be driven by individual #1 who performed the most dives. Dive shape analysis revealed that most PrCAs were concentrated in three types of dive clusters (2, 4, and 5). This result is in line with former studies demonstrating that dive shapes provide relevant information on the seal diving activity (Davis et al., 2003, 2013). This suggests that lactating females partition their underwater activities (hunting, exploring, etc.) between different types of dives, with specific types of dives during which they would feed. Given they seem to hunt occasionally as opportunistic feeders (Davis et al., 2003, 2013; Heerah et al., 2014), these foraging dives would not necessarily be dedicated solely to feeding. PrCAs that took place during classified dives were concentrated in three dive clusters (2, 4, and 5). Cluster 2 dives are characterized by high sinuosity during the whole dive, suggesting feeding events can occur anytime during the dive. Dives in cluster 4 are characterized by a V shape and a reduced sinuosity during the descent phase, supporting the hypothesis that females dive directly to the seafloor where they might encounter their prey. They seem to select the shortest and optimal route to get to their prey (King & Marshall, 2022). The skewness on the ascent phase suggests higher sinuosity, likely representing hunting events. Indeed, previous studies observed that Weddell seals display higher vertical sinuosity during hunts, with lower vertical instantaneous velocity (Fuiman et al., 2007; Heerah et al., 2014). However, this result (prey searching during the ascent phase) is in opposition with former work on the McMurdo Sound colony, where Fuiman et al. (2007) observed sinuous descent and straight ascent during foraging dives in spring. Those patterns were clearly observed in cluster 3 dives during which no PrCA was recorded, characterized by a sinuous track during the descent phase. The straight ascent phase of those dives seems to indicate that females swim directly to the surface, probably because they were reaching their physiological dive limits. The third cluster with PrCAs (cluster 5) is characterized by a W shape and very high vertical sinuosity at the scale of the whole dive, underlining the importance of movement sinuosity to detect hunting events, rather than bottom time (Heerah et al., 2014). Other dive classification studies often assume square‐shaped dives (such as cluster 1 dives) are foraging dives, since from an optimal foraging perspective benthic predators should maximize time spent at the seafloor, where they hunt (Austin et al., 2006; King & Marshall, 2022; Schreer et al., 2001). Our results are not in line with these studies: Here, we demonstrated that benthic hunting dives were better characterized by rapid descent phase, high vertical sinuosity during the ascent and V or W shapes. This could be associated with the shallow bathymetry of the small area used by females during lactation. Regarding other dive clusters, the type of behaviors/activities (social, territorial, etc.) associated with these dives remain unknown and further research is needed to identify their functions. However, the results emerging from the use of these dive metrics must be taken with caution: A recent study from Allegue et al. (2023) tested several track and dive‐based metrics on Southern elephant seals and found that most of the movement and track metrics taken alone were unable to correctly depict feeding activities. They suggest that single dive metrics such as the bottom time might not provide a sufficiently accurate description of the foraging behavior to predict the number of prey encountered. The use of such metrics combined with other biologging technologies and sonar data in this study could open new perspectives for a more accurate description of foraging behavior. The functional principal component analysis suggests a similar number of dive clusters observed in this study as have been observed in previous studies. Most of these studies detected four dive clusters (Hindell et al., 2002; Shero et al., 2018) and used a limited number of dive descriptors to run statistical analysis (Davis et al., 2013; Godard et al., 2020; Hindell et al., 2002; Schreer et al., 2001; Shero et al., 2018). Madden et al. (2008) delineated five dive shapes, using 18 descriptors, and demonstrated that three of the five dive shapes were characteristic of foraging dives. The differences with this study might be due to the focus on lactating females, and to the analysis of the dive profile as a whole curve instead of a series of finite descriptors.

#### 4.2.2 Constraints due to the presence of the pup and reproductive strategy

The presence of the pup‐constrained females' dives and their ability to forage. Pups are totally dependent on their mother to feed and gain energy; moreover, they have to remain on top of sea ice at the beginning of their life as they do not have enough fat to thermoregulate in the water and follow their mother's dives. We observed pups exploring under‐ice habitat in video footage despite differing aerobic capacity from the mother, as observed by Burns et al. (1997), Kikuchi et al. (2010) and Sato, Mitani, Naito, and Kusagaya (2003). The mother was often carrying the pup on her head or back to help it swim. While alone underwater, mothers were observed returning regularly to the sea‐ice platform to check up on their pup, consequently reducing their foraging effort. Lactating females are often found within the fast ice region associated with stable platforms, far from open water areas, so the pup, which is still unable to dive deep or thermoregulate (in the first few days after its birth), will be safe during the whole lactating period (Burns, 1999; Kikuchi et al., 2010). Therefore, ice holes constitute the only access to water for mothers during this period. Davis et al. (2013) demonstrated there are different dive strategies according to the types of dives, categorized between free‐ranging and ice hole restricted dives. Ice hole dives might be constrained by the need to return to an ice hole to breathe and the impossibility to return to the surface whenever needed. These lactating females also demonstrated much shallower dives than usual observations in the same area for post‐molt Weddell seals, as a response to the presence of the pup remaining on the sea‐ice platform (Heerah et al., 2013). Mean dive depth during the post‐molt period is usually around 280 m, with a maximum at 904 m (Heerah et al., 2013). In addition to diving more shallowly, lactating females spent much less time diving than post‐molt Weddell seals (more than 50% of their time diving per day; Shero et al., 2018).

Phocids reduce foraging effort during lactation and can lose up to 30% of their body mass during this period (Shero et al., 2018). Lactation duration might therefore be influenced in part by the amount of fat reserve that the mother can invest in pup nursing. Among seals, the proportion of fat stores before giving birth ranges from 24.3% in harbor seals to 40.2% in Weddell seals (Bowen et al., 1992; Wheatley et al., 2006b). Lactation duration among seals can therefore be placed along a continuum, with Weddell seals having the longest known lactation interval (Costa & Maresh, 2022; Wheatley et al., 2008). This can be explained by their ability to resort to a mixed capital‐breeding strategy, supporting the cost of lactation both through their body reserves and foraging. Interindividual variability among female Weddell seals regarding the position on the capital‐to‐income‐breeding spectrum has been described in previous works, depending on the age, the lactation period, or the female's condition (Hill, 1987; Hindell et al., 2002; Sato et al., 2002). Consequently, the time pups need to spend nursing to put on enough energy reserves before weaning differs. However, females globally spend more time foraging as they get later into the lactation period, progressively reducing the frequency and duration of nursing: Tedman and Bryden (1979) described that nursing can go from about 15 suckling events per 24 h at the beginning of lactation within the first week (totaling 5 h per day), to eight suckling events per 24 h (3.5 h per day) on the sixth week. As they get deeper into lactation period, pups spend more time in the water and their energetic needs for swimming increase with their maneuvering skills underwater. Therefore, the amount of energy used by pups increases with time, while less energy is stored, resulting in potential pups' mass loss. No quantification of such mass loss is available in the literature to the best of our knowledge; however, reduction of pup mass gain rate in the course of the lactation has been documented at McMurdo and Hutton Cliffs, East Antarctica (Tedman & Bryden, 1979; Wheatley et al., 2008).

Our results provide additional evidence that female Weddell seal still forage during lactation, as suggested in Testa et al. (1989), Sato et al. (2002), Wheatley et al. (2008), Costa and Maresh (2022), Shero and Burns (2022), and Foster‐Dyer et al. (2023). The small number of PrCA and the very reduced foraging area observed during the lactation period suggest that the mother is constrained to feed close to the location where she leaves her pup on the sea‐ice platform, therefore local prey abundance may be a decisive factor of reproductive success (Costa & Maresh, 2022). This mixed strategy, between 100% capital and 100% income strategy, guarantees that reproductive success is not solely dependent on the mother's ability to build reserves or on local prey availability: Females can either fast if the environmental conditions are temporally unfavorable for hunting, or forage on available prey if their body reserves are not sufficient (Costa & Maresh, 2022). This work sheds light on previously unexplored aspects of foraging behavior, such as shallow water environments, targeting benthic prey and generally focusing on single prey items rather than schools, and evidence of visual scanning through observed head movements.

It must be emphasized that the limited number of individuals in this study prevents us from making assumptions on the energy budget of female Weddell seals during lactation. Moreover, prey availability within the pup area may limit opportunities to find schools of prey.

### 4.3 Benthic dives

Almost all feeding events detected in our study were benthic (98% of overall PrCAs). This is congruent with former studies hypothesizing that Weddell seals feed on bentho‐pelagic prey during summer (Casaux et al., 2009) and during lactation (Hindell et al., 2002), as well as during other parts of their life cycle (Goetz, 2015; Plötz et al., 2001), rather than on epipelagic prey, such as *Pleuragramma antarcticum* (Plötz et al., 2001). Conversely, studies conducted during seasons other than summer demonstrate that seals tend to feed in midwater, rather than benthically (Fuiman et al., 2007). Studies conducted in McMurdo Sound demonstrated that Weddell seals' diet is dominated by Antarctic silverfish (*Pleuragramma antarcticum*), captured at the midwater depth, and small notothenioids (*Pagothenia borchgrevinki*, *Trematomus* sp.) (Burns et al., 1998; Fuiman et al., 2007; Goetz et al., 2017). La Mesa et al. (2022) demonstrated in another region of East Antarctica (Terra Nova Bay) that the coastal area is dominated by notothenioids fish, with most species being benthic at adult stage. These species might therefore be a part of Weddell seals' diet in Terre Adélie as well. Weddell seals are also a predator of Antarctic toothfish, especially in the food web near shore in the Ross Sea. However, there is still debate regarding the importance of the Antarctic toothfish in the seals diet: While isotopic analyses tend to demonstrate low contribution of the species to the seals diet (1.6% of overall diet), older and larger seals seem to be more dependent on this type of prey (up to 19% of overall diet) (Goetz et al., 2017). The sonar tags deployed here provide insights into prey characteristics, giving valuable clues to draw hypotheses on the type of prey encountered. From the echo size, it is unlikely that juvenile or adult toothfish were predated by Weddell seals in this study, and to the best of our knowledge, no observations of toothfish have been reported on the continental shelf near Dumont D'Urville, Terre Adélie. It would be interesting in the future to compare Weddell seal and Antarctic toothfish prevalence and distribution to assess whether Weddell seals' foraging area in Terre Adélie is linked to Antarctic toothfish populations, both as competitors and prey (Ainley et al., 2020).

Despite females diving at shallower depths during lactation compared to post‐molt dives (average of 280 m depth, Heerah et al., 2013), lactating females still performed PrCAs during their deepest dives (mean diving depth: 36 m, mean PrCA depth: 88 m). Primary production is expected to be high within melting sea ice in summer, thus phytoplankton blooms underneath the ice may attract prey at the sea ice/water interface (Arrigo, 2016). The depth difference with other parts of their life cycle is probably due to general shallow diving behavior during lactation, and to sufficient prey availability on the seafloor close to the lactating location.

### 4.4 Fine‐scale predation interactions in link with prey ecology

This study is among the first to describe prey reactions to foraging seals (Goulet et al., 2019). Benthic dives of Weddell seals were often associated with prey reactions. We observed that most of prey identified by the sonars departed from the seafloor when the seal approached. Weddell seals in our study therefore likely hunt their prey by chasing them off the seafloor, a behavior that has been discussed in previous works such as in Heerah et al., 2013, but has never directly to the best our knowledge. Although this observation cannot lead to prey identification, it provides interesting insights into Weddell seals' diet. Notothenioids, building nests on the seafloor, appear to dominate the coastal region of Terra Nova bay (East Antarctica), as demonstrated by La Mesa et al. (2022): the previous observation supports the hypothesis that Weddell seals might feed on this type of prey. Seals search for prey during the approach phase by turning their head left and right. This observation supports Heerah et al. (2014) suggesting that Weddell seals are generalist foragers and opportunistic predators reacting to the sudden appearance of prey (Heerah et al., 2014). Surprisingly, while the approach strategy of the two lactating females appeared to be opportunistic, they seemed to have used an archetypal capture pattern, turning their head left most of the time when the prey reacted. This suggests that seals position themselves on the left of their prey and catch them from the left. The sample size in this study is however too small to infer any archetypal behavior that could suggest handedness, as Friedlaender et al. described in other marine mammals such as the blue whales (Friedlaender et al., 2017). Gliding patterns assessed from acceleration data were observed during the approach phase as well as during the catching phase, as also observed in former studies on their fine‐scale foraging ecology (Fuiman et al., 2007).

## 5 CONCLUSION

This study provides new insights into the fine‐scale foraging ecology of lactating female Weddell seals. Our results provide additional evidence that female Weddell seals still forage during lactation, and shed light on previously unexplored aspects of foraging behavior. The findings enhance our understanding of how females adjust their foraging activity in response to the constraints imposed by the lactation of their pup. Prey capture attempts were mostly on single, benthic prey, probably hidden among rocks and sediments on the seafloor. Their foraging area was restricted, compared with foraging trips during other parts of their annual cycle. Our observations also demonstrated how they partition their dives according to different underwater activities: Some dives were associated with feeding, while other were not. Sonar tags have therefore proven to be an effective tool to describe marine predators' foraging strategy and predator–prey interactions at a very fine scale.

## AUTHOR CONTRIBUTIONS

**Adélie Antoine:** Data curation (equal); formal analysis (equal); investigation (equal); methodology (equal); visualization (equal); writing – original draft (lead); writing – review and editing (equal). **Sara Labrousse:** Conceptualization (lead); data curation (lead); formal analysis (lead); funding acquisition (lead); investigation (lead); methodology (lead); supervision (lead); validation (lead); visualization (lead); writing – original draft (lead); writing – review and editing (lead). **Pauline Goulet:** Conceptualization (lead); data curation (lead); methodology (lead); resources (lead); software (lead); writing – review and editing (lead). **Mathilde Chevallay:** Formal analysis (equal); investigation (equal); methodology (equal); writing – review and editing (equal). **Joris Laborie:** Resources (equal); writing – review and editing (equal). **Baptiste Picard:** Data curation (equal); investigation (equal); software (equal); writing – review and editing (equal). **Christophe Guinet:** Methodology (equal); supervision (equal); writing – review and editing (equal). **David Nerini:** Formal analysis (equal); investigation (equal); methodology (equal); validation (equal); visualization (equal); writing – review and editing (equal). **Jean‐Benoît Charrassin:** Conceptualization (equal); funding acquisition (lead); project administration (lead); supervision (lead); validation (equal); writing – review and editing (equal). **Karine Heerah:** Funding acquisition (lead); methodology (equal); project administration (lead); resources (lead); supervision (lead); validation (lead); writing – review and editing (equal).

## FUNDING INFORMATION

This work was supported by the French Polar Institute (IPEV) program 1182 ASSET, the “Sentinel of the Sea‐Ice Environment” SENSEI BNP‐Paribas Foundation project, and the French National Center for Space Studies CNES‐TOSCA program “Weddell seals bio‐oceanographers of the Antarctic sea‐ice” (P.I.: J.B. Charrassin and S. Labrousse). Adelie Antoine received a PhD fellowship for the GDR OMER (CNRS). Karine Heerah received fundings from a Marie Sklodowska Curie fellowship for this project (project 792042 – FEAST – H2020‐MSCA‐IF‐2017).

## CONFLICT OF INTEREST STATEMENT

The authors declare that they have no competing interests.

## Supporting information


Appendix S1–S5.

## Data Availability

All data, code, and materials used in the analyses will be available on a Dryad repository upon acceptance, https://doi.org/10.5061/dryad.jm63xsjj0.
