# Supplementary material for: Beneath the Antarctic sea‐ice: Fine‐scale analysis of Weddell seal (Leptonychotes weddellii) behavior and predator–prey interactions, using micro‐sonar data in Terre Adélie
Source: Ecol Evol. 2023 Dec 11;13(12):e10796. doi: 10.1002/ece3.10796 (PMC10714067; doi:10.1002/ece3.10796)
Supplement: Supplementary file 1 — Appendix S1–S5. [file ECE3-13-e10796-s001.docx]

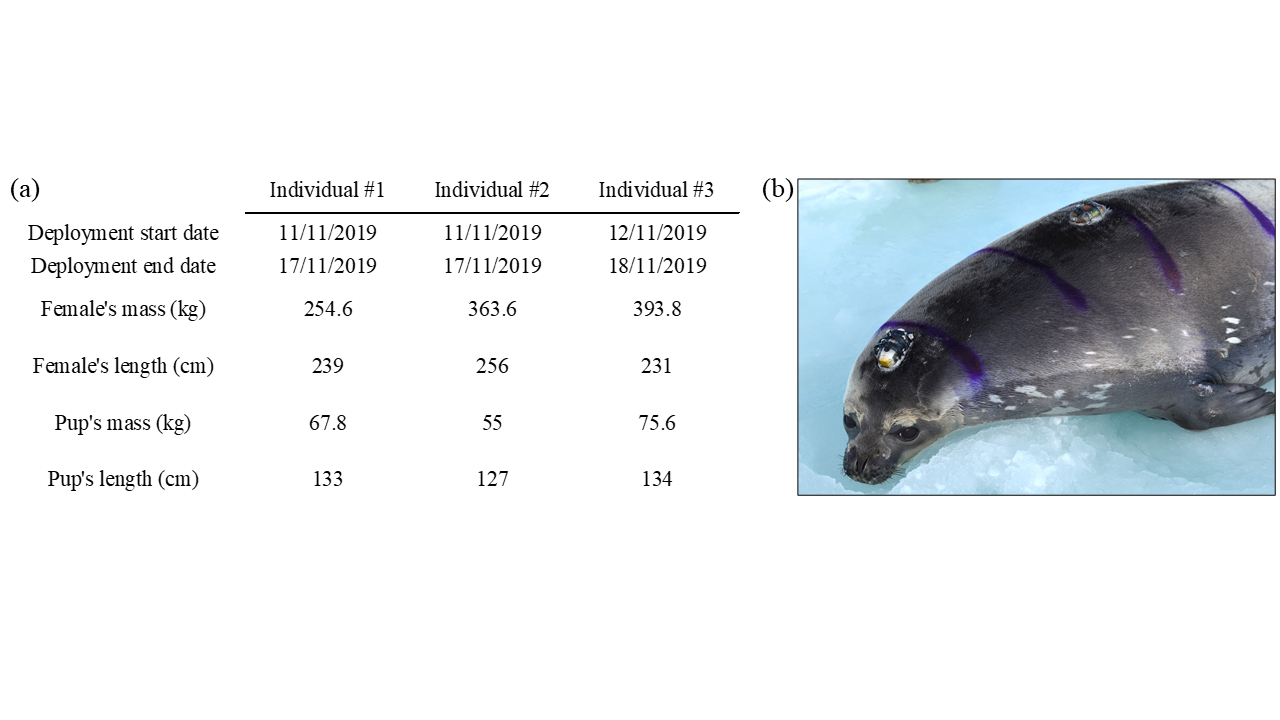
**Supplementary file S1**

General information on the deployment dates, characteristics of the studied individuals and their pup (panel (a)). On the left (panel (b)), photography of an equipped seal, with the micro-sonar on the head, and the GPS tag on the back. (Photography: courtesy of Karine Heerah).


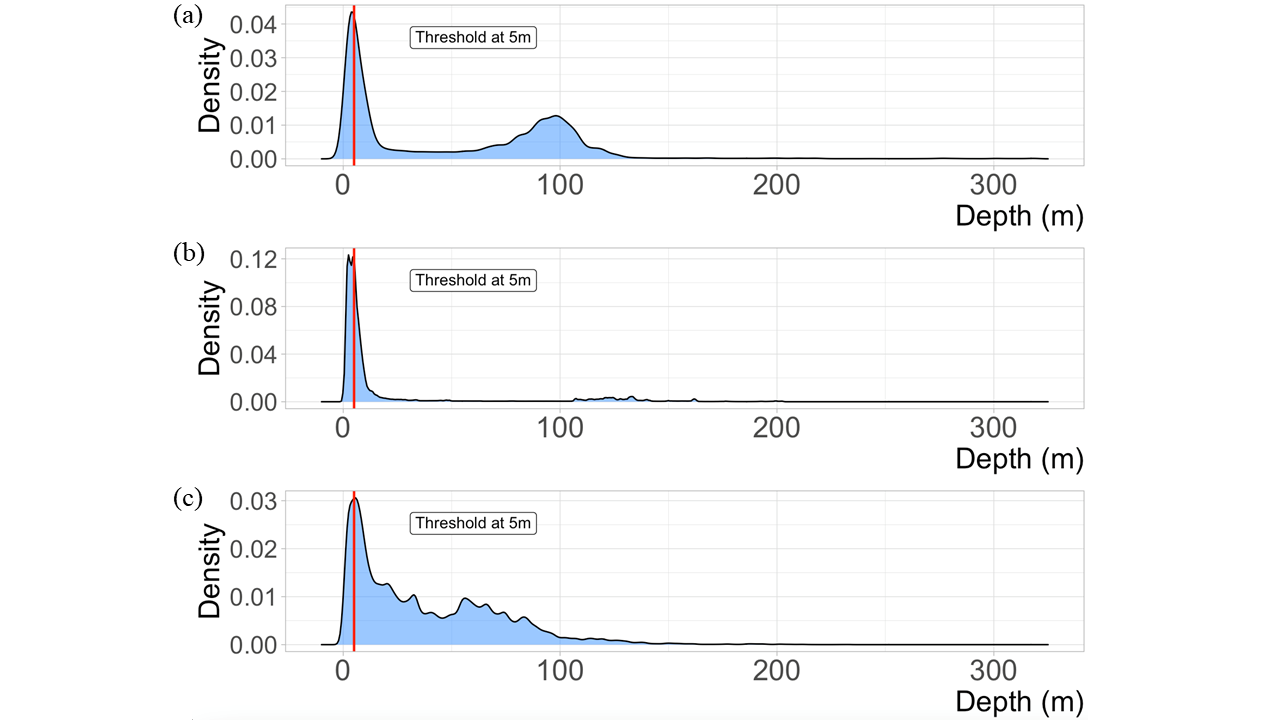
**Supplementary file S2**

Dive depth density distribution plots of individual 1 (panel (a)), 2 (panel (b)) and 3 (panel (c)). The threshold, set at 5m to distinguish proper dives from other underwater activities, is indicated by the red line.

**
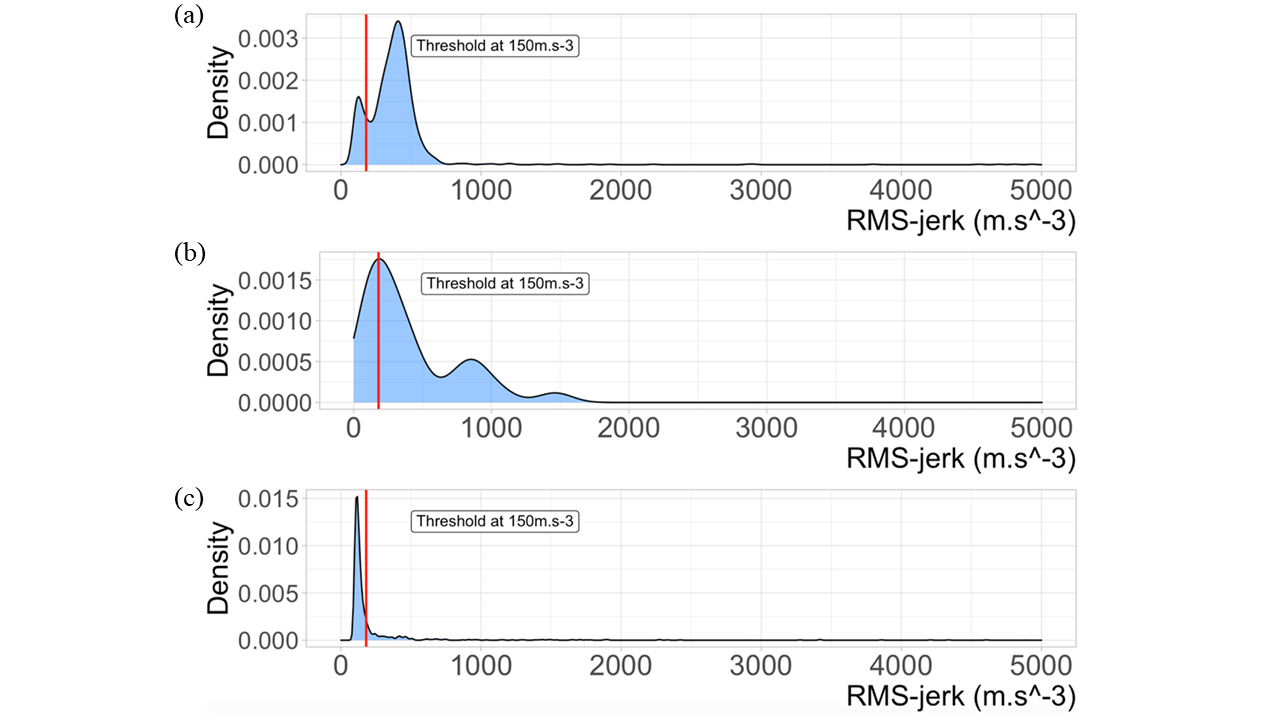
Supplementary file S3**

RMS-jerk density distribution plots of individual 1 (panel D), 2 (panel E) and 3 (panel F). The threshold, set at 150m.s^-3^, is indicated by the red line.

**
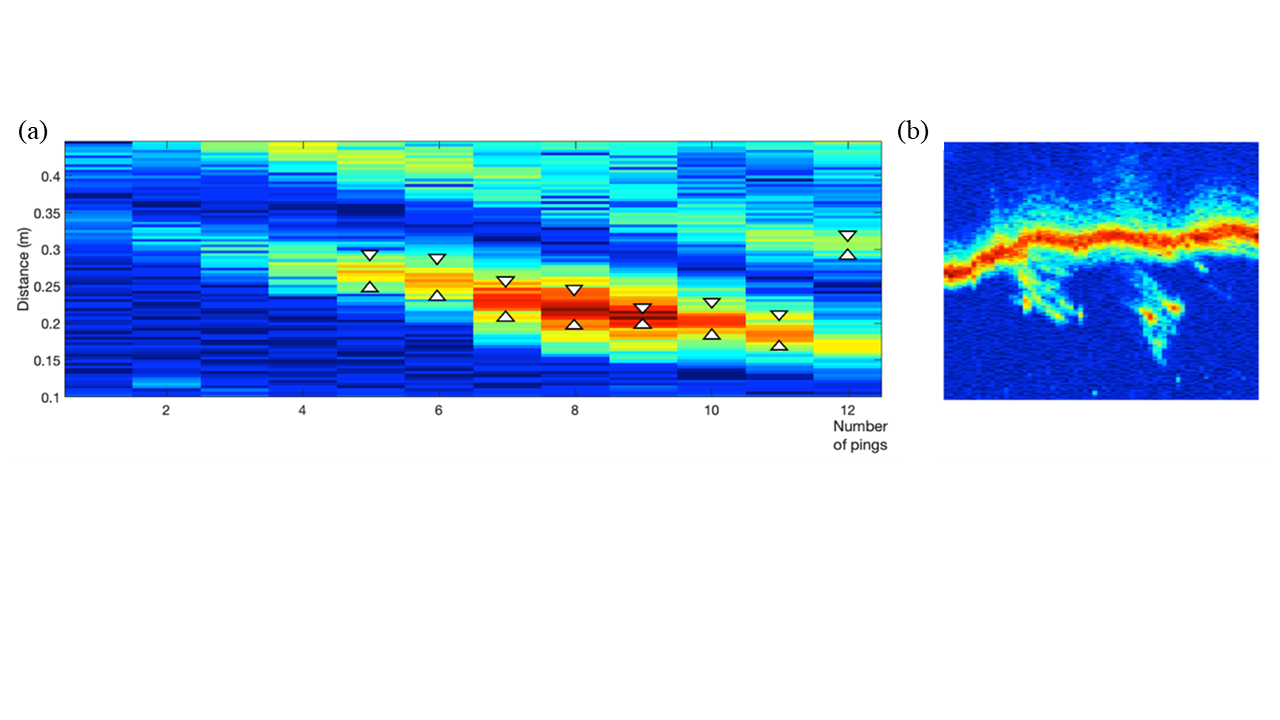
Supplementary file S4**

Illustration of the acoustic size computation (panel (a)). Pings are composed of stacked pixels, colored according to the echo-to-noise ratio. Based on the contrast of colors in each ping, the boundaries of the prey trace are defined. Given each pixel represent an acoustic size of 4mm, the acoustic size of the prey is computed by counting the number of pixels included within the prey trace’s boundaries, using the ping with the best contrast between the prey trace and the background noise. On the left (panel (b)), example of several signals in one ping, probably a school of prey.

**Supplementary file S5**

Two-to-two comparison of tested clustering methods. c-RI: corrected rand index. VI: variation of information index.
